# Evaluation of an immunoassay for determination of plasma efavirenz concentrations in resource-limited settings

**DOI:** 10.7448/IAS.17.1.18979

**Published:** 2014-06-05

**Authors:** Alemseged Abdissa, Lubbe Wiesner, Helen McIlleron, Henrik Friis, Åse Bengård Andersen, Pernille Kæstel

**Affiliations:** 1 Department of Medical Laboratory Sciences & Pathology, Jimma University, Jimma, Ethiopia; 2 Department of Infectious Diseases, Odense University Hospital, Odense, Denmark; 3 Division of Clinical Pharmacology, Department of Medicine and Institute of Infectious Diseases and Molecular Medicine, University of Cape Town, Cape Town, South Africa; 4 Department of Nutrition, Exercise and Sports, University of Copenhagen, Copenhagen, Denmark

**Keywords:** efavirenz, immunoassay, TDM, antiretroviral therapy, method evaluation, LC–MS/MS

## Abstract

**Introduction:**

Therapeutic drug monitoring (TDM) may improve antiretroviral efficacy through adjustment of individual drug administration. This could result in reduced toxicity, prevent drug resistance, and aid management of drug–drug interactions. However, most measurement methods are too costly to be implemented in resource-limited settings. This study evaluated a commercially available immunoassay for measurement of plasma efavirenz.

**Methods:**

The immunoassay-based method was applied to measure efavirenz using a readily available Humastar 80 chemistry analyzer. We compared plasma efavirenz concentrations measured by the immunoassay with liquid chromatography tandem mass spectrometry (LC-MS/MS) (reference method) in 315 plasma samples collected from HIV patients on treatment. Concentrations were categorized as suboptimal<1 µg/ml, normal 1–4 µg/ml or high>4 µg/ml. Agreement between results of the methods was assessed via Bland-Altman plot and κ statistic values.

**Results:**

The median Interquartile range (IQR) efavirenz concentration was 2.8 (1.9; 4.5) µg/ml measured by the LC–MS/MS method and 2.5 (1.8; 3.9) µg/ml by the immunoassay and the results were well correlated (ρ=0.94). The limits of agreement assessed by Bland–Altman plots were −2.54; 1.70 µg/ml. Although immunoassay underestimated high concentrations, it had good agreement for classification into low, normal or high concentrations (*K*=0.74).

**Conclusions:**

The immunoassay is a feasible alternative to determine efavirenz in areas with limited resources. The assay provides a reasonable approximation of efavirenz concentration in the majority of samples with a tendency to underestimate high concentrations. Agreement between tests evaluated in this study was clinically satisfactory for identification of low, normal and high efavirenz concentrations.

## Introduction

Efavirenz is an essential part of current first-line antiretroviral combination therapies for the management of HIV in patients either infected with HIV alone or co-infected with tuberculosis [1–3]. However, effectiveness depends on attaining adequate systemic concentrations to suppress viral replication with minimal adverse effects.

Beside adherence to the regimen and dietary and drug interactions, genetically determined differences in drug distribution and elimination may significantly affect systemic concentrations [4]. For example, CYP2B6 polymorphisms, which explained inter-individual variability in efavirenz concentration remarkably [5], occur more predominantly in Africans (up to 50% of the population) [6]. High inter-individual variability in plasma concentrations of most antiretroviral drugs, and the lower virologic failure rates and adverse events when optimal concentrations are achieved, have already been demonstrated [7,8]. Thus, therapeutic drug monitoring (TDM) might aid optimization of antiretroviral therapy (ART) [9,10], particularly in Africa where genetic variability is high; but its application depends on the availability of reliable methods appropriate for low-income settings.

Several analytical methods have been developed to quantify the non-nucleoside analogue reverse transcriptase inhibitors (NNRTI) in human plasma, but in most cases high performance liquid chromatography (HPLC) with ultraviolet or fluorescence detection, liquid chromatography–tandem mass spectrometry (LC–MS/MS) or capillary electrophoresis are used [11]. These methods use a complicated and time-consuming sample pre-treatment and instrument set-up. In addition, the equipment is expensive and requires highly skilled operators. This makes them unsuitable in resource-constrained countries.

Automated immunoassay methods have been developed by ARK diagnostics (Sunnyvale, CA, USA) for determination of plasma efavirenz, nevirapine and protease inhibitors. The aim of this study was to apply the immunoassay-based method on a readily accessible chemistry analyzer and to evaluate the agreement with efavirenz concentrations determined by the immunoassay using LC-MS/MS as a reference.

## Methods

This study was conducted on plasma samples collected from 230 HIV-infected patients who were taking efavirenz-based antiretroviral treatment at Jimma University Hospital, Ethiopia. In addition, 85 efavirenz-free plasma samples collected from participants on a nevirapine-based therapy in the same study setting were included. This was to determine the discrimination power of the method between positive and negative samples, and to set the lowest detection limit of the immunoassay method. Thus, a total of 315 plasma samples were analyzed. All participants provided consent and the study was approved by the Jimma University ethics committee and national research ethics committee of Ethiopia.

One month after initiation of treatment, blood samples were collected into Ethylenediaminetetraacetic acid (EDTA) tubes in the morning, approximately 12 hours after the evening 600 mg dose of efavirenz. Plasma was prepared from blood samples by centrifugation at 2000 g for 10 minutes and stored at −70°C until shipment to Cape Town on dry ice for efavirenz concentration determination using LC–MS/MS, or analysis using the Humastar 80 chemistry analyzer (Human Diagnostics, Wiesbaden, Germany) in Jimma, Ethiopia.

### Liquid chromatography–tandem mass spectrometry

LC–MS/MS was considered a gold standard for the validation of the immunoassay method. Efavirenz concentrations were determined by LC–MS/MS in the laboratory of the Division of Clinical Pharmacology, University of Cape Town. The assay was validated according to the US Food and Drug Administration (FDA) [12] and the European Medicines Agency (EMA) [13] guidelines. Plasma samples were extracted with acetonitrile. Isocratic chromatography was performed on a Luna 5 µm PFP (2), 100 A, 50 mm×2 mm analytical column using acetonitrile and 0.1% formic acid (1:1, v/v) as mobile phase at a flow rate of 350 µl/min. An AB Sciex API 4000 mass spectrometer was operated at unit resolution in the multiple reaction monitoring (MRM) mode, monitoring the transition of the protonated molecular ions at m/z 316.1 to the product ions at m/z 244.0 for efavirenz, and monitoring the transition of the protonated molecular ions at m/z 320.2 to the product ions m/z 246.1 for the stable isotope labelled efavirenz internal standard. The calibration curve fitted a quadratic (weighted by 1/concentration 2) regression over the ranges 0.0195–20.0 µg/ml. The laboratory participates in the International Interlaboratory Control Program of Stichting Kwaliteitsbewaking Klinische Geneesmiddelanalyse en Toxicologie (Hague, The Netherlands) and the AIDS Clinical Trial Group (ACTG), Pharmacology Quality Control Program.

### Efavirenz immunoassay

Plasma efavirenz concentrations were determined by the immunoassay “ARK EFV-Test™” (ARK Diagnostics Sunnyvale, CA, USA) using the automated Humastar 80 analyzer (Human Diagnostics, Wiesbaden, Germany). The assay is a homogeneous immunoassay intended for *in vitro* diagnostic use to quantify efavirenz in human serum or plasma. When sample and reagents are mixed, a drug in the sample competes with a drug labelled by the enzyme glucose-6-phosphate dehydrogenase (G6PDH) for antibody binding sites. Enzyme activity decreases upon binding to the antibody so that the drug concentration in the sample can be measured in terms of enzyme activity. Active enzyme converts nicotinamide adenine dinucleotide (NAD) to NADH, resulting in an absorbance change that is measured spectrophotometrically at 340 nm. The NADH absorbance is directly proportional to drug concentration in the sample. Endogenous serum G6PDH does not interfere because the coenzyme functions only with the bacterial enzyme (from *Leuconostoc mesenteroides*) used in the assay. Calibration curves were prepared in each run based on six standards of known concentrations of efavirenz (0–12 µg/ml). Precision of the immunoassay was determined by measuring three levels of efavirenz control materials (ARK Diagnostics) and pooled serum (efavirenz-free) in each run (*n*=8). Samples with immunoassay values higher than the highest standard concentration (8 µg/ml) were diluted 1:2 with distilled water and reanalyzed.

### Statistical analysis

Raw optical density (OD) values were exported in text file format and calibration curves generated (logit OD against ln efavirenz concentration) using STATA/IC version 11.2 (StataCorp LP, College Station, USA). Spearman’s rank correlation coefficient and linear regression were calculated to directly compare data obtained from the immunoassay and the LC–MS/MS. Wilcoxon signed-rank test was used to assess the difference in median obtained by the two methods. A Bland–Altman plot with 95% limits of agreement (mean difference±2 SD) was constructed to visualize the agreement between the methods [14]. Furthermore, measurements were classified based on the established normal reference interval for efavirenz trough concentration in <1 µg/ml (suboptimal), 1–4 µg/ml (normal therapeutic range) and >4 µg/ml (high concentration) [15,16] and kappa statistics was calculated to assess agreement in classification. Precision of the immunoassay was expressed as the coefficient of variation (CV%) for between-run assessment.

## Results

The relative accuracy of the immunoassay was evaluated by comparison to the efavirenz concentrations determined by LC–MS/MS in 230 plasma samples from participants who took efavirenz. The median (IQR) efavirenz was 2.8 (1.9; 4.5) µg/ml by the LC–MS/MS and 2.5 (1.8; 3.9) µg/ml by the immunoassay method. Wilcoxon signed-rank test indicated that the medians of the values obtained by the two methods were different (*p*<0.0001). The range of concentrations obtained by the immunoassay was 0.11–15.1 µg/ml compared to 0.01–16.6 µg/ml with LC–MS/MS. Linear regression analysis found a 0.744 ug/ml increase in efavirenz concentration determined by the immunoassay for each 1 ug/ml increase in efavirenz concentration determined by LC-MS/MS (*y=*0.4844+0.7437X). The Spearman’s correlation coefficient indicated a very good positive correlation (ρ=0.94).

**Figure 1:**
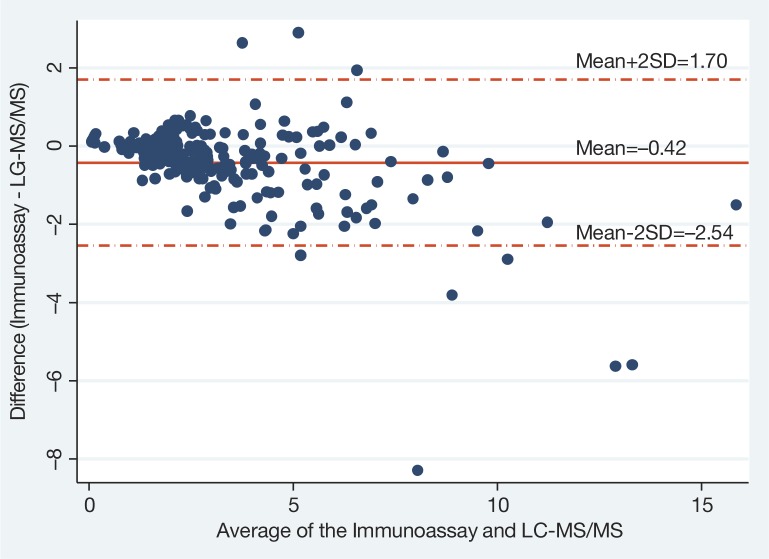
Bland–Altman plots of the agreement between the immunoassay and the LC–MS/MS. The solid line is the mean difference between the two methods (Immunoassay-LC–MS/MS); the dashed lines are 95% limits of agreement (±2 SD).

A Bland–Altman plot was constructed to evaluate the agreement between the immunoassay and the LC–MS/MS (Figure 1), which underlines poorer agreement at higher values. The mean efavirenz concentration determined by the immunoassay was 0.42 µg/ml lower than that of LC–MS/MS and the 95% limits of agreement between the two methods were −2.54 to 1.70 µg/ml, and the range of the limit of agreement was 4.24 µg/ml. This means that 95% of differences between the two methods were within these values. The immunoassay agreed with the LC–MS/MS within 1 µg/ml in 189/230 (82.2%) of the samples, overestimated by>1 µg/ml in 5/230 (2.2%) of samples and underestimated by >1 µg/ml in (36/230) 15.6% of samples. Among the samples with underestimated efavirenz concentration, 31/36 (86%) were above 4 µg/ml and 9/36 (25%) were above 8 µg/ml.

The mean difference was calculated for each concentration category and it was noted that the range was broad in the high category in contrast to that of negative, suboptimal and normal ranges (Table 1).

**Table 1:** Mean difference (Immunoassay – LC–MS/MS) between the efavirenz concentrations obtained by immunoassay and LC–MS/MS in different categories

Category from LC–MS/MS	Number of samples	Mean difference	95% CI of difference
Negative	85	0.31	−0.97; 1.59
Suboptimal (<1 µg/mL)	22	0.08	−0.4; 0.56
Normal (1–4 µg/mL)	136	−0.15	−1.29; 0.99
High (>4 µg/mL)	72	−0.72	−4.15; 1.97

The immunoassay correctly classified 20/22 (91%) of the suboptimal efavirenz concentrations, 130/136 (96%) of the normal and 49/72 (68.1%) of the high efavirenz concentrations (Table 2). Overall, the method agreement for classification in efavirenz concentrations into suboptimal, normal and high was considered “good” for immunoassay versus LC–MS/MS (agreement 86.5% of observations, *k*=0.74). However, 23 out the 72 high efavirenz concentrations were misclassified as normal.

**Table 2:** Cross tabulation between the immunoassay and the LC–MS/MS in categorization of the efavirenz concentration, *n* (%)

	Immunoassay			
		
LC–MS/MS	Suboptimal (<1 µg/mL)	Normal (1–4 µg/mL)	High (>4 µg/mL)	All
Negative	81 (95.3)	2 (2.3)	2 (2.3)	85 (100)
Suboptimal (<1 µg/mL)	20 (90.9)	2 (9.1)	0 (0.0)	22 (100)
Normal (1–4 µg/mL)	2 (1.5)	130 (95.6)	4 (2.9)	136 (100)
High (>4 µg/mL)	0 (0.0)	23 (31.9)	49 (68.1)	72 (100)

The mean efavirenz concentration of 85 efavirenz-free samples measured by the immunoassay was 0.32±0.6 µg/ml. Thus, while the immunoassay classified most efavirenz-free samples in the suboptimal category, 4.7% (*n*=4) were classified as having efavirenz concentrations in the normal range. Omitting these four samples with extreme values (1.1–4.3 µg/ml) the mean (range) of concentrations was 0.2 (0.1–0.3) µg/ml. Among the efavirenz negative samples, 16 (18.8%) were above the lower limit of quantification (LLQ) set by the manufacturer at 0.25 µg/ml.

We tested the precision of the immunoassay by measuring repeatedly (*n*=8) the controls provided by the manufacturer with the actual efavirenz concentration, which ranged from 1 to 8 µg/ml. In addition, efavirenz negative sample was used (Table 3). The CV% of the controls ranged from 6.0 to 10%; however, the efavirenz negative sample had relatively high variation, with a CV% of 20 (Table 3).

**Table 3:** Between-run precision, coefficients of variation and accuracy (% of value provided by ARK diagnostics) of efavirenz concentrations measured by the immunoassay (*n*=8 runs)

	Actual value in µg/ml	Mean ±SD µg/ml	CV%	Accuracy
Control low	1	1.42±0.14	9.9	142
Control medium	4	4.61±0.34	7.4	115
Control high	8	8.31±0.50	6.0	104
Efavirenz negative sample	0	0.15±0.03	20.0	

Eight samples determined by the immunoassay to have concentrations>8 µg/ml were reanalyzed using immunoassay after diluting 1:2 with distilled water. With the undiluted samples, the mean difference between the immunoassayed concentrations and those determined using LC–MS/MS was −2.45 µg/ml and the 95% limits of agreement were −6.77; 1.87 µg/ml. Dilution, however, improved both the mean difference (−0.55 µg/ml) and the 95% limits of agreement (−3.35; 2.25 µg/ml). Regarding classification in the categories, the test under evaluation correctly classified high values (>8 µg/ml) without dilution of the samples; thus dilution did not bring any difference with respect to classification into concentration categories.

## Discussion

We evaluated a low cost efavirenz quantification method requiring minimal expertise and no sample pre-treatment. The complexity of these operations is about the same as other blood chemistry analysis and can be applied on a number of clinical chemistry analyzers without additional training. The method demonstrated good performance to discriminate suboptimal, normal and high efavirenz concentrations.

Although TDM for antiretroviral drugs is not yet a routine practice in many countries, guidelines proposing specific indications and optimal therapeutic ranges are already established for some drugs including for efavirenz 1–4 µg/ml [17,18]. However, lack of simple and low cost assays is a bottleneck for implementation of TDM in low resource countries where HIV is rampant. The urgent need for such an assay in sub-Saharan Africa was the primary motivation of the current study.

This study demonstrated a mild discrepancy of values in the efavirenz concentration, with the immunoassay underestimating the efavirenz concentration, particularly at high concentrations. However the immunoassay correctly classified the majority of samples into suboptimal, normal and high concentration.

The immunoassay exhibited good precision with CV% of the controls ranging from 6.0 to 9.9%. The low precision (CV%= 20) with the serum pool was anticipated as it was free from efavirenz and the detection limit of the assay is higher, 0.25 µg/ml. However, the absolute variation of efavirenz in the efavirenz-free samples was lower than samples with measurable efavirenz. In the current study, the efavirenz concentrations measured by immunoassay were<0.33 µg/ml in the vast majority of efavirenz-free samples. Thus, it may be reasonable to increase the LLQ of this method on the Humastar chemistry analyzer to 0.4 µg/ml, from 0.25 µg/ml as suggested by the manufacturer [19].

Efavirenz has a concentration–response relation that makes it attractive for TDM to predict therapeutic efficacy. For instance, patients having an efavirenz trough plasma concentration less than 1.1 µg/ml have been associated with virologic failure [20]. In addition, neuropsychiatric adverse effects, such as insomnia, have been associated with efavirenz concentrations above 4 µg/ml [17,21]. Thus, this immunoassay may not be able to detect patients with potential adverse effects due to the underestimation of high concentrations. However, the clinical utility of this test method is acceptable in light of the fact that TDM goes beyond the identification of patients with sub-therapeutic or excessive drug concentrations because a “normal” or close to normal drug concentration can be extremely valuable in ruling out the extreme conditions or providing reassurance that a dose is correct in the face of a possible treatment failure or toxicity [22].

This study also investigated the benefit of diluting plasma samples with high efavirenz concentration, to compensate for its poor performance at high concentrations. Dilution of samples with high concentration helped to obtain closer mean value to that of the reference method, relatively small mean difference and comparable 95% limits of agreement to the entire undiluted samples. Thus, we recommend diluting samples to verify high concentrations if actual concentrations are required.

An important advantage of the immunoassay is that it can be implemented using various chemistry analyzers, as in this case the Humastar 80. The Humastar 80 has been widely distributed in Ethiopia as part of the expansion of standard HIV service delivery to monitor drug induced adverse effects associated with ART (personal communication). Thus, it will be a feasible strategy to perform TDM of antiretroviral drugs in resource-constrained settings, like Ethiopia. Recently, a similar immunoassay was suggested as an attractive alternative to HPLC analyses for determinations of nevirapine concentrations in breast milk [23].

The strengths of this study include the large sample covering a wide range of concentrations. The potential limitation is that we did not perform reproducibility analysis on patients’ samples using the immunoassay. However, we believe that precision of efavirenz in samples would not differ markedly from that of controls and thus it only represents a small limitation to this work.

## Conclusions

In summary, the immunoassay is appropriate for the low-cost assessment of efavirenz concentrations. This assay represents a major step towards effective TDM for antiretroviral drugs in resource-limited settings where the conventional methods of the clinical laboratory are unavailable or prohibitively expensive. The immunoassay provided acceptably accurate results in the suboptimal and normal concentrations compared to LC–MS/MS. The observed difference at high concentrations can be improved by diluting samples with high concentration if the actual values are needed. Agreement between tests evaluated in this study was clinically satisfactory for identification of suboptimal, normal and high efavirenz concentration.
